# Maternal determinants of healthy infant feeding practices in Oman: an analytical epidemiology study

**DOI:** 10.3389/ijph.2026.1609924

**Published:** 2026-10-29

**Authors:** Basma Al Yazeedi, Omar Al Omari, Salma Al Yazeedi, Atika Khalaf

**Affiliations:** 1 College of Nursing, Sultan Qaboos University, Muscat, Oman; 2 Oman College of Health Sciences - North Sharqiyah Branch, Ibra, Oman; 3 Hogskolan Kristianstad, Kristianstad, Sweden; 4 Mohammed Bin Rashid University of Medicine and Health Sciences, Dubai, United Arab Emirates

**Keywords:** health beliefs, infant feeding practices, infant nutrition, maternal adherence, MENA region

## Abstract

**Objectives:**

This study investigated the predictors of infant feeding. The focus was on understanding how maternal sociodemographic, psychosocial, and childbirth factors influence infant feeding practices.

**Methods:**

An analytical cross-sectional study was conducted between October 2023 and March 2024, using a validated Infant Feeding Practices and Beliefs questionnaire. Mothers of children aged one to 3 years were recruited from 120 healthcare centers across nine Governorates of Oman. Descriptive, inferential, and multivariable linear regression analyses were conducted, with the significance level set at less than 0.05.

**Results:**

The study analyzed 3,036 surveys. The findings suggested that healthier beliefs, receipt of feeding counselling, and high awareness are associated with better infant feeding practices. Baccalaureate education or more and family income of at least 1000 OMR showed negative influence on practices, although were positively associated with the beliefs. Cesarean delivery was associated with poorer adherence to feeding practices.

**Conclusion:**

This study offers insights into the factors associated with infant feeding, emphasizing culturally informed health promotion strategies. Comprehensive infant feeding counseling shows promise in improving adherence to infant feeding recommendations.

## Introduction

Optimal and comprehensive infant feeding practices are critical for enhancing public health outcomes. Nutritional intake during the first year of life significantly impacts both short-term and long-term health [1]. Global issues like childhood stunting and overweight further emphasize the need for improved infant feeding practices. In Oman, the prevalence of stunting among children under 5 years is approximately 12.9% in 2024, while overweight in this age group has reached 8.8% [2].

Healthy infant feeding practices support growth, immunity, and cognitive development. Exclusive breastfeeding significantly enhances an infant’s immunological defenses [3]. Globally, exclusive breastfeeding for the first 6 months can prevent over 800,000 child deaths annually and reduce the risk of infections and chronic diseases [4]. However, only 48% of infants are exclusively breastfed worldwide [5], with factors such as insufficient maternity protection, aggressive marketing of substitutes, and inadequate healthcare training contributing to poor adherence. In Oman, the prevalence of exclusive breastfeeding for the first 6 months is only 23.2% [6], indicating substantial gaps relative to global targets and necessitating updated national evidence on the determinants of suboptimal practices.

The introduction of solid foods is recommended at 6 months of age [7], aligning with the maturation of digestive processes necessary for effective food processing [8]. For that, early complementary feeding raises the risk of infant mortality due to diarrhea and pneumonia [4]. Concurrently, honey consumption is discouraged during the first year due to the risk of infant botulism [9], likewise egg whites due to their association with food allergies [10].

Infant feeding practices are primarily shaped by underlying beliefs. Understanding these beliefs is essential, as they significantly influence caregivers’ behavior and decision-making. These beliefs are deeply rooted in cultural practices, maternal perceptions, and societal influences [11, 12]. They can affect practices such as the introduction of prelacteal feedings, avoidance of colostrum, and perceptions of infant satiety and weight [13, 14]. Maternal age, education, and acculturation also play significant roles in shaping feeding decisions [15], influencing infant health, growth, and long-term eating habits. Understanding these complex beliefs is crucial for providing culturally sensitive and effective support for infant feeding.

While initiatives like the Baby-Friendly Hospital project exist in Oman, inconsistent implementation presents challenges [16]. Adherence to WHO global guidelines is low [17], primarily due to sociocultural misconceptions, work constraints, and limited support [18, 19]. Modernization, lifestyle shifts, and bottle-feeding trends, particularly in urban settings, further threaten infant feeding practices [20]. Traditional advice from elder relatives often promotes early use of herbal drinks or solid foods, viewing formula as modern or superior [21]. Despite higher education levels being associated with better breastfeeding practices, employment and aggressive formula marketing can undermine this trend [22]. Increased maternal employment without workplace lactation support contributes to inadequate breastfeeding practices and early weaning [23]. Also, significant gaps remain in postnatal support and the implementation of the Baby-Friendly Hospital Initiative, especially in rural areas [24]. Oman currently lacks detailed, updated data on infant feeding. National surveys, such as the Oman Multiple Indicator Cluster Survey, provide an overview but fail to explore socio-cultural drivers, regional disparities, or maternal barriers to optimal practices [25].

This study assessed mothers’ adherence and beliefs toward infant feeding recommendations and examined the associating demographic, psychosocial, and childbirth predictors, among a diverse sample of mothers from multiple governorates in Oman. The findings enhanced the understanding of infant feeding practices determinants and their public health implications, informing further research on effective interventions and national strategies tailored to improve infant nutrition, growth, and long-term health outcomes in Oman and neighboring countries.

## Methods

### Study design

The study used an analytical cross-sectional design within an epidemiological framework to assess maternal adherence to infant feeding recommendations and investigate associated maternal demographic, psychosocial, and childbirth predictors. The study design was considered suitable because it facilitated the exploration of national practices regarding infant feeding while examining their association with different factors [26].

### Setting

A diverse sample of mothers from multiple governorates in Oman was conveniently recruited from health centers between October 2023 and March 2024. Health centers are governmental facilities affiliated with the Ministry of Health and provide a free, safe, and accessible setting for health services [27]. With a national vaccination rate of 99% for children younger than 3 years [28], health centers were deemed a suitable setting for reaching the study participants.

The healthcare centers involved were located in varied geographical settings, including urban and rural areas, which provided access to mothers from diverse community settings. This variation often corresponds to sociodemographic factors, which increases heterogeneity of the sample.

This study gathered data from the nine largest governorates in Oman (from a total of 11 governorates), which include the majority of the population attending Ministry of Health primary care services. These governorates encompassed 29 cities, where 120 health centers were approached for data collection. The large number of recruited health centers was intended to reduce facility-level selection bias and to capture geographic variation in practices.

### Participants

A convenience sampling technique was used to recruit the study participants. The study involved mothers of children aged one to 3 years. Since the focus is on actual infant feeding practices, it was important for mothers to provide a comprehensive report of their practices during their child’s first year of life. Furthermore, mothers of children older than 3 years may have difficulty recalling their infant feeding practices. Eligible participants were mothers who could read and write in Arabic, since the questionnaire was in Arabic, and who provided informed consent. Exclusion criteria included mothers with a cognitive disability affecting their understanding of the survey, non-Omani mothers since the study aimed to understand the practices from a cultural perspective, and mothers who had already answered the survey for another child.

A total of 28 research assistants (RAs) were recruited and trained to follow a standardized data collection protocol. According to the protocol, RAs attended health centers during morning shifts, where they approached and greeted potential participants for a quick eligibility screening. Mothers who met the inclusion criteria were invited to participate in the study and handed the consent form, which included detailed information about the study on its front page. After obtaining consent, the RAs provided a data collection form and remained on-site to assist and address any questions raised by the participants.

The sample size was calculated using multivariable linear regression analysis, focusing primarily on the effects of individual predictors. Following the guidelines set by Maxwell [29], and assuming a small effect size (R^2^ = 0.02), a two-sided significance level of 0.05, and a statistical power of 80%, a minimum of 403 participant is required. However, we aimed to recruit 0.5%–1% of the child population in each governorate [30], supporting the study’s epidemiological framework (Supplementary Material 1). Efforts were made to enhance diversity by recruiting from varied geographic regions and a large number of health centers across Oman. Data collection continued at health centers within each governorate until the target sample size was achieved or no additional participants could be recruited.

### Study variables

The study’s primary outcome was IFP reflected as the maternal adherence to infant feeding recommendations. The studied maternal demographic factors included age, education, parity, and family income. The maternal psychosocial factors included infant feeding beliefs (IFB), receipt of infant feeding counseling, and self-reported infant feeding knowledge level. We also investigated potential confounders relative to childbirth history, including birth status, mode of delivery, and Neonatal Intensive Care Unit (NICU) admission.

### Study instrument

The researchers used a validated Arabic Infant Feeding Practices and Beliefs descriptive questionnaire developed by Al Yazeedi et al. (2025). The 54-item questionnaire has two main sections. The first section includes 34 items addressing sociodemographic characteristics (11 items), birth and clinical history (11 items), and infant feeding history during the first year (12 items). The second section contained 20 items designed to provide a comprehensive assessment of infant feeding practices and beliefs.

The IFP section includes 10 items, most of which were answered using multiple-choice options. Mothers were asked about various IFP based on the WHO infant feeding recommendations [31]. The first item assessed exclusive breastfeeding during the first 6 months, with responses recorded on a five-point Likert scale ranging from “never” to “always.” The second item asked about breastfeeding continuation through the first year, with responses recorded as “yes” or “no.” The remaining items addressed the timing of introducing formula milk, complementary foods, animal protein, 100% fresh juice, yoghurt, egg, unmodified milk, and honey. The third to tenth items offered four age-range options in months as response choices. Responses to the ten items were then categorized and scored as meeting = 1 or not meeting = 0 the WHO infant feeding recommendations, resulting in a total IFP score ranging from 0 to 10 [32]. The first item responses were sorted as follows: 1 = always, 0 = mostly, sometimes, rarely, and never [31].

Following the IFP section, there were 10 four-point Likert-scale items addressing infant feeding beliefs, with response options: strongly disagree, disagree, agree, and strongly agree. The ten items related to infant feeding beliefs were similar to those in the IFP section, but were written to focus on beliefs.

The questionnaire’s validity was established through face and content validation methods. Pilot testing demonstrated acceptable test-retest reliability r = 0.73 [p = 0.007] and r = 0.65 [p = 0.005] for IFP and IFB, respectively. Further details about the development of the questionnaire are published in another manuscript [32].

### Ethical approval

Researchers secured ethical approval from the Research and Ethics Committee at the College of Nursing, Sultan Qaboos University (CON/IG/2022/9) and the Health Studies and Research Approval Committee at the Ministry of Health (MOH/CSR/23/26906). The data collection front page included information about the study and a consent statement. In addition, anonymity was maintained in the study as no identifying information, such as name, date of birth, or medical record number, was collected. Data were stored in a password-protected computer, and paper-based questionnaires were stored in a locked cabinet inside a locked office.

### Data analysis

Data were entered into the SPSS program version 23, checked for accuracy, and cleaned, resulting in the removal of surveys with ≥20% missing data. The maternal demographic characteristics, infant feeding counseling, and infant feeding knowledge, besides childbirth history variables, were analyzed using frequency and percentages, while means and standard deviations were reported for IFP and IFB. Percentages were calculated using the number of non-missing responses for each variable.

Inferential statistics, including Pearson correlation, were used to examine associations between IFP and IFB. Independent t-tests were used to assess differences between IFP scores across demographic characteristics, infant feeding counseling, childbirth status, delivery mode, and NICU. One-way ANOVA was used to compare IFP scores across infant feeding knowledge categories (high, moderate, low). Multivariable linear regression analysis was run to assess the significant predictors. The assumption of normality, linearity, homoscedasticity, and multicollinearity was assessed before running the analysis. The significance level was set at p < 0.05.

## Results

A total of 3563 surveys were collected with a response rate of 91%. However, 308 surveys were completed for children under 12 months, while an additional 219 surveys did not answer all 10 IFP items, leading to their exclusion. Ultimately, we analyzed a total of 3,036 surveys, which included 2,907 complete IFB responses.

### Participants characteristics

Table 1 presents the participants’ characteristics. Mothers were predominantly 18–35 years old (n = 2,218, 73.1%) and had less than a baccalaureate-level education (n = 1986, 65.4%). Most had one to three children (n = 1954, 64.4%), and a monthly family income under 1,000 Omani Riyals (∼2,596 USD) (n = 1966, 64.8%). Less than half received infant feeding counseling (n = 1,312, 43.2%), and 53.4% (n = 1,621) reported moderate infant feeding knowledge.

**TABLE 1:** Participants’ characteristics (Maternal Determinants of Healthy Infant Feeding Practices in Oman: An Analytical Epidemiology Study. Oman. 2026).

Variables	Mean	SD
IFP score (N = 3036)	6.36	1.72
IFB score (N = 2,907)	28.91	2.95
​	n	%
Maternal demographic		
Mother’s age		
18–35 years	2,218	73.1
36–55 years	789	26.0
Missing	29	1.0
Mother’s education		
Less than baccalaureate	1986	65.4
Baccalaureate or more	1,047	34.5
Missing	3	0.1
Parity		
1–3	1954	64.4
More than 3	1,040	34.3
Missing	42	1.4
Family income		
Less than 1,000	1966	64.8
1,000 or more	1,035	34.1
Missing	35	1.2
Maternal psychosocial		
Recipient of infant feeding counseling		
No	1,686	55.5
Yes	1,312	43.2
Missing	38	1.3
Reported infant feeding knowledge		
High	1,179	38.8
Moderate	1,621	53.4
Low	221	7.3
Missing	15	0.5
Childbirth history		
Child gender		
Male	1,651	51.3
Female	1,530	47.5
Missing	38	1.2
Child age (at time of study)		
12–17 months	1,046	34.5
18–23 months	882	29.1
24–29 months	877	28.9
30–36 months	231	7.6
Missing	0	0
Birth status		
Full-term	2,612	86.0
Premature/Post-term	377	12.4
Missing	47	1.5
Delivery mode		
Vaginal	2,307	76.0
Cesarean section	723	23.8
Missing	6	0.2
NICU admission		
No	2,799	92.2
Yes	225	7.4
Missing	12	0.4

IFP, infant feeding practices; IFB, infant feeding beliefs; NICU, neonatal intensive care unit.

Majority of children were distributed across three age groups during the time of data collection: 34.5% (12–17 months), 29.1% (18–23 months), and 28.9% (24–29 months). The sex distribution was almost even (51.3% male; 47.5% female). Most were born full-term (n = 2,612, 86.0%), via vaginal delivery (n = 2,307, 76.0%), and without NICU admission (n = 2,799, 92.2%).

### Description of IFP and IFB

Table 2 presents the frequencies and percentages of IFP and IFB, highlighting several discrepancies between practices and beliefs. The results suggested that the practice of exclusive breastfeeding during the first 6 months was much lower than the belief (35.8% versus 93.5%). Additionally, while 85.7% believe it is healthy to introduce complementary foods at 6 months of age, 68.8% report actually practicing it. Similarly, 82.5% of mothers perceive the introduction of yogurt after 6 months as a healthy practice, but only 62.6% actually do so. Secondary analysis showed that many introduce yogurt either too early (19.6% before 6 months) or too late (14.1% after the child turns 1 year), with 27.1% introducing solids between four and 5 months (Supplementary Material 2). Moreover, the analysis shows that there are generally less favorable beliefs associated with introduction of fresh juice (36.2%) and infant formula (58.8%), which align with the observed feeding practices. Interestingly, the percentages for favorable fresh cow’s milk and honey practices were higher than the reported beliefs, at 48.2% and 52.3%, respectively, as shown in Table 2.

**TABLE 2:** Frequency and percentage of favorable infant feeding practices and beliefs (Maternal Determinants of Healthy Infant Feeding Practices in Oman: An Analytical Epidemiology Study. Oman. 2026).

Items	IFP (N = 3036)	IFB (N = 2,907)
	n (%)	n (%)
Q1. Exclusive breastfeeding in the first 6 months	1,086 (35.8)	2,840 (93.5)
Q2. Breastfeeding in the first year	2,683 (88.4)	2,892 (95.3)
Q3. Infant formula after 6 months	1,660 (54.7)	1785 (58.8)
Q4. Solid foods after 6 months	2090 (68.8)	2,602 (85.7)
Q5. Meat, chicken, and fish after 6 months	2,595 (85.5)	2,264 (74.6)
Q6. 100% fresh unsweetened juice after first year	1,077 (35.5)	1,099 (36.2)
Q7. Yogurt after 6 months	1902 (62.6)	2,506 (82.5)
Q8. Egg after 6 months	1,587 (52.3)	2,309 (76.1)
Q9. Fresh cow’s milk after first year	2,326 (76.6)	1,462 (48.2)
Q10. Honey after first year	2,310 (76.1)	1,589 (52.3)

### Factors associating with IFP and IFB scores

Table 1 shows that the mean IFP score was 6.36 (SD = 1.72) and the mean IFB score is 28.91 (SD = 2.95). Below are details related to the bivariate analysis with maternal demographic, maternal psychosocial, and childbirth history factors.

#### Maternal demographic factors

The independent t-test indicated that mothers with less than a baccalaureate degree had a significantly higher mean IFP score (mean = 6.43, SD = 1.72) than those with higher education levels (mean = 6.24, SD = 1.73; t = 2.89, p = 0.004). Interestingly, the trend reversed for the IFB, with mothers with higher education reporting more favorable beliefs (t = −6.59, p < 0.001). Similarly, higher family income significantly affected IFB scores (t = −5.50, p < 0.001). However, mothers with a monthly income of less than 1,000 OMR reported significantly higher mean IFP scores than those with higher household incomes (t = 2.45, p = 0.02).

Results revealed no significant association between mother’s age and IFP scores (t = −1.13, p = 0.26) or IFB scores (-t = 0.67, p = 0.50). Additionally, the results indicated no differences in the IFP or IFB scores between mothers with three or fewer children and those with more children (t = −1.52, p = 0.13 and t = 1.11, p = 0.27, respectively) Further details are presented in Table 3.

**TABLE 3:** Bivariate analysis for Infant Feeding Practices (IFP) and Infant Feeding Beliefs (IFB) scores based on the Maternal Demographics, Maternal Psychosocial Factors, and Childbirth History (Maternal Determinants of Healthy Infant Feeding Practices in Oman: An Analytical Epidemiology Study. Oman. 2026).

Variables	IFP			IFB		
	Mean (SD)	t/F/r	p	Mean (SD)	t/F/r	p
Maternal Demographic						
Mother’s age	​	−1.13	0.26	​	−0.67	0.50
18–35 years	6.35 (1.74)	​	​	28.88 (2.97)	​	​
36–55 years	6.43 (1.65)	​	​	28.96 (2.89)	​	​
Mother’s education	​	2.89	0.004	​	−6.59	<0.001*
Less than baccalaureate	6.43 (1.72)	​	​	28.64 (2.83)	​	​
Baccalaureate or more	6.24 (1.73)	​	​	29.41 (3.11)	​	​
Parity	​	−1.52	0.13	​	1.11	0.27
1–3	6.32 (1.72)	​	​	28.95 (2.91)	​	​
4 or more	6.42 (1.74)	​	​	28.82 (3.01)	​	​
Family income	​	2.45	0.02*	​	−5.50	<0.001*
Less than 1,000	6.42 (1.75)	​	​	28.68 (2.81)	​	​
1,000 or more	6.26 (1.66)	​	​	29.33 (3.13)	​	​
Maternal psychosocial						
IFB score	​	0.29	<0.001	​	​	​
Infant feeding counseling	​	−4.21	<0.001	​	−4.88	<0.001
No	6.24 (1.76)	​	​	28.68 (2.98)	​	​
Yes	6.51 (1.67)	​	​	29.22 (2.89)	​	​
Infant feeding knowledge	​	21.16	<0.001	​	17.04	<0.001
High	6.56 (1.67)	​	​	29.11 (2.98)	​	​
Moderate	6.28 (1.73)	​	​	28.94 (2.92)	​	​
Low	5.92 (1.79)	​	​	27.67 (2.78)	​	​
Childbirth history						
Birth status	​	2.79	0.01*	​	0.70	0.48
Full-term	6.40 (1.73)	​	​	28.93 (2.93)	​	​
Pre/Post-term	6.14 (1.66)	​	​	28.81 (3.06)	​	​
Delivery mode	​	2.42	0.02	​	0.05	0.96
Vaginal	6.41 (1.72)	​	​	28.91 (2.95)	​	​
Cesarean section	6.23 (1.73)	​	​	28.91 (2.96)	​	​
NICU admission	​	2.47	0.01	​	−0.30	0.77
No	6.38 (1.73)	​	​	28.90 (2.95)	​	​
Yes	6.09 (1.66)	​	​	28.96 (2.94)	​	​

* Equal variances not assumed with Levene’s test p < 0.05. IFP, infant feeding practices; IFB, infant feeding beliefs; NICU, neonatal intensive care unit.

#### Maternal psychosocial factors

A positive correlation emerged between IFP and IFB scores (r = 0.29, p < 0.001). Mothers without infant feeding counseling had lower IFP scores (mean = 6.24, SD = 1.76) than counseled mothers (mean = 6.51, SD = 1.67; t = −4.21, p < 0.001). Likewise, mothers who had received infant feeding counseling reported significantly higher mean IFB scores compared to those without counseling history (t = −4.88, p < 0.001). Moreover, ANOVA revealed statistically significant differences in IFP and IFB scores across infant feeding knowledge groups (F = 21.16, p < 0.001; F = 17.04, p < 0.001, respectively) See Table 3.

#### Childbirth history

Analysis revealed that mothers who had full-term birth scored significantly better IFP compared to those who had pre or post term delivery (t = 2.79, p = 0.01). However, the birth status did not differ significantly based on the IFB scores (t = 0.70, p = 0.48). Additionally, vaginal delivery associated with significantly higher IFP scores (mean = 6.41, SD = 1.72) than cesarean section (mean = 6.23, SD = 1.73; t = 2.42, p = 0.02). The case was not the same for the IFB scores (t = 0.05, p = 0.96). NICU admission history had a statistically significant influence on the IFP (t = 2.47, p = 0.01), but not the IFB (t = −0.30, p = 0.77), as illustrated in Table 3.

### Associated factors of IFP and IFB scores

A multivariable linear regression analysis was conducted for both IFP and IFB. Cases with missing values for any variables included in the regression analysis were excluded through listwise deletion. Ultimately, the analysis model included 2,723 participants for both IFP and IFB.

The IFP multivariable linear regression analysis was statistically significant (p < 0.001) and accounted for 11% of the variance (Adjusted R^2^ = 0.108). This analysis identified several independent predictors of IFP scores. Healthier beliefs about infant feeding correlated with higher scores (B = 0.17, p < 0.001). Mothers who received counseling on infant feeding demonstrated higher scores (B = 0.17, p = 0.01). Conversely, maternal education of at least a baccalaureate degree and a family income of at least 1000 OMR were linked to lower scores (B = −0.23, p = 0.001 and B = −0.21, p = 0.004, respectively). Mothers with moderate or low infant feeding knowledge exhibited poorer practices compared to those with high knowledge level (B = −0.23, p < 0.001 and B = −0.34, p = 0.01, respectively). Mothers who delivered via cesarean section had lower IFP scores (B = −0.18, p = 0.02). However, mother’s age, parity, birth status, and NICU admission were not significant predictors to IFP (see Table 4).

**TABLE 4:** Multivariable linear regression analysis of Infant Feeding Practices (IFP) and Infant Feeding Beliefs (IFB) scores with regression coefficients (Maternal Determinants of Healthy Infant Feeding Practices in Oman: An Analytical Epidemiology Study. Oman. 2026).

Variables	IFP			IFB		
	B	SE	p	B	SE	p
(Constant)	1.59	0.32	<0.001	28.61	0.13	<0.001
Mother’s age	0.08	0.08	0.32	0.028	0.14	0.84
Mother’s education	−0.23	0.07	0.001	0.559	0.13	<0.001
Parity	0.05	0.08	0.47	−0.13	0.13	0.33
Family income	−0.21	0.07	0.004	0.41	0.13	0.001
IFB	0.17	0.01	<0.001	-	-	-
Infant feeding counseling	0.17	0.06	0.01	0.48	0.11	<0.001
Moderate infant feeding knowledge	−0.23	0.07	<0.001	−0.20	0.12	0.09
Low infant feeding knowledge	−0.34	0.13	0.01	−1.35	0.23	<0.001
Birth status	−0.17	0.10	0.09	−0.12	0.18	0.49
Delivery mode	−0.18	0.08	0.02	−0.04	0.13	0.77
NICU	−0.21	0.13	0.10	0.09	0.22	0.70

IFP, infant feeding practices; IFB, infant feeding beliefs.

IFP, model (N = 2,723)*: R* ^*2*^ = .112, Adjusted *R* ^*2*^ = .108, *F* = 31.12, *p* < 0.001. Outcome: IFP, scores.

IFB, model (N = 2,723): *R* ^*2*^ = .039, Adjusted R^2^ = .035, F = 10.98 P < .001. Outcome: IFB, scores.

Reference categories: mother’s age = 35 or younger, mother’s education = less than baccalaureate, parity = 1–3, family income = less than 1000 OMR, infant feeding counselling = no, moderate infant feeding knowledge = high, low infant feeding knowledge = high, birth status = full term, delivery mode = vaginal, NICU = no.

For the IFB model, the multivariable linear regression was also statistically significant (p < 0.001) and accounted only for 4% of the variance (Adjusted R^2^ = 0.035). Significant predictors included maternal education of at least a baccalaureate degree (B = 0.56, p < 0.001), family income equal to 1000 OMR or more (B = 0.41, p = 0.001), and receipt of infant feeding counseling (B = 0.48, p < 0.001). Furthermore, low infant feeding knowledge was associated with a decrease in IFB scores (B = −1.35, p < 0.001).

## Discussion

Infant feeding patterns are shaped by a complex interplay of regional practices and household dynamics, suggesting population-specific determinants to optimal practices. This study’s findings indicate that while Omani mothers believe in the principles of healthy infant feeding, these beliefs do not always translate into corresponding practices, revealing challenges in implementing infant feeding recommendations. The study revealed key determinants to infant feeding practices, contributing to a model for guiding further research and targeted interventions as illustrated in Figure 1. This study supports the premise that healthier infant feeding practices are associated with healthier beliefs, counseling recipients, and greater awareness.

**FIGURE 1:**
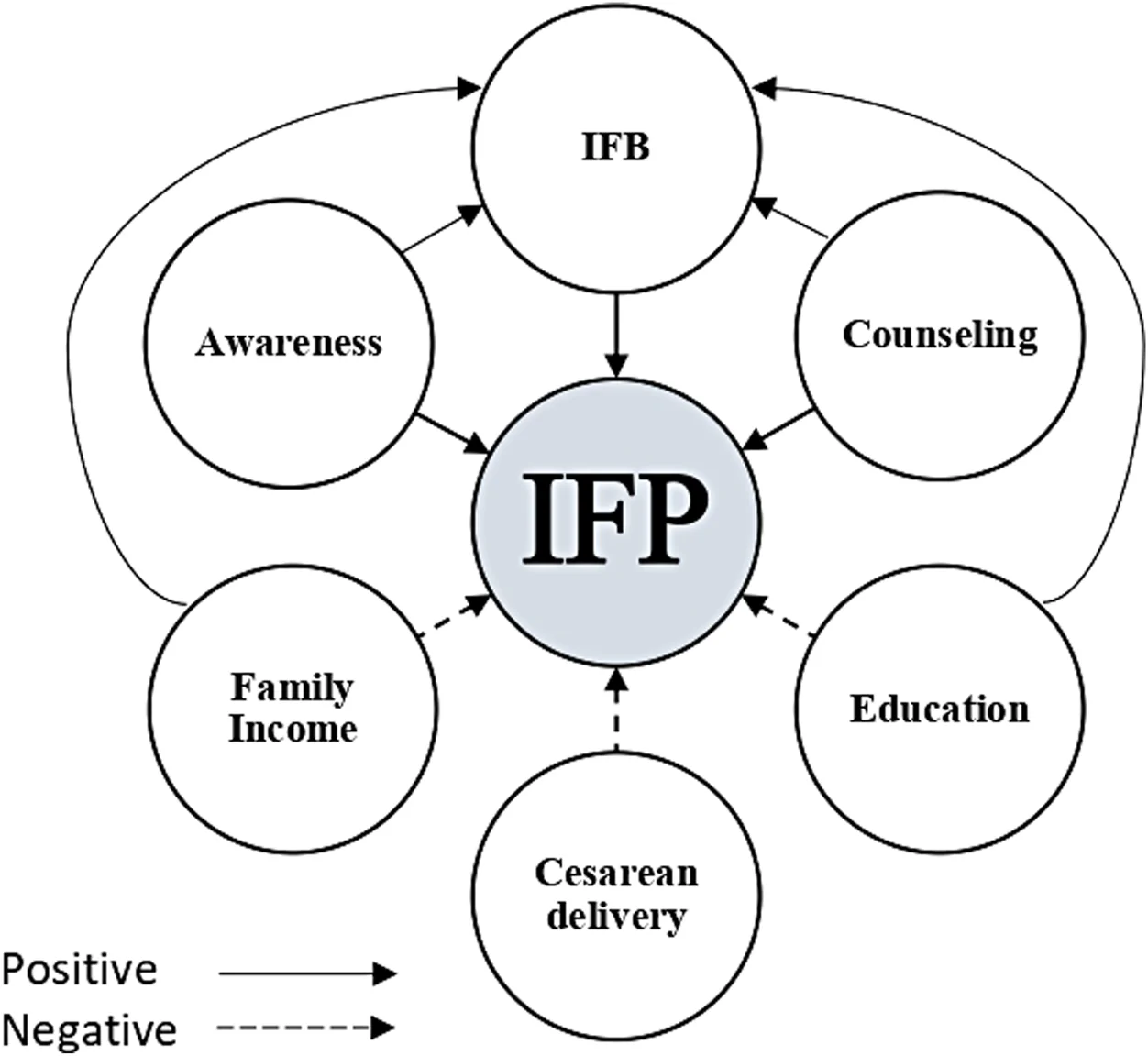
Model of factors associated with infant feeding practices (Maternal Determinants of Healthy Infant Feeding Practices in Oman: An Analytical Epidemiology Study. Oman. 2026).

Mothers’ positive beliefs stem from access to knowledge on infant feeding and a supportive social environment [33]. Research highlights that increased health knowledge among parents is essential for promoting better dietary practices, enabling mothers to make informed decisions [34]. Consistent with this premise, this study found that mothers who received counseling were more likely to endorse healthy infant feeding practices. This echoes the findings of Yenit, Genetu [35], who demonstrated that infant feeding counseling reduced the prevalence of prelacteal feeding, giving a newborn substances other than breast milk before breastfeeding begins, in Ethiopia. Counseling also serve as a protective factor against misinformation and cultural misconceptions that often compromise child nutrition. In the Nigerian context, research has reported that counseling was a strong predictor of exclusive breastfeeding even among HIV-positive mothers [36], underscoring its relevance across diverse health and social conditions.

Nevertheless, this study found more favorable beliefs toward breastfeeding practices and less favorable beliefs toward complementary foods, such as fresh juice, fresh cow’s milk, and honey. Similar pattern was observed in Turkey, where only 37% of infants start solid foods at the recommended age of 6 months, despite optimistic breastfeeding practices [37]. This implies that counseling services may have focused on breastfeeding rather than comprehensive guidance on complementary feeding, potentially overlooking other important, risk-related infant feeding practices.

This study suggests that maternal age is not associated with infant feeding practices, contradicting a trend consistent with findings from several countries in the MENA region, including Egypt, Jordan, Palestine, and Sudan [38]. Prior research linked advanced age to better feeding practices, noting that older mothers tend to possess greater childbearing experience, knowledge, and confidence [17, 38]. Nonetheless, this study findings suggested that parity is not linked to healthier feeding practices, nor the age to better feeding beliefs. Future research may focus on contemporary risks associated with the younger-mother generation, such as the effects of social media [39, 40].

Although this study found that mothers with higher levels of education and income generally held healthier beliefs about infant feeding, a contrasting trend emerged in actual infant feeding practices, suggesting that mere beliefs are insufficient for adopting healthy practices. This inconsistency could be explained by factors related to job commitments and maternity leave policies [41]. In the GCC nations, it is not uncommon for socioeconomic advancement and increased workforce participation among educated women to coincide with inadequate infant feeding practices. [42]. For instance, the Oman National Nutrition Survey revealed that only 23.2% of infants under 6 months are exclusively breastfed, where inflexible workplaces identified as a main challenge for educated mothers [43, 44]. Research from low- and middle-income countries showed that families with greater financial resources may prematurely introduce suboptimal foods due to misconceptions about the superiority of formula feeding [45]. This underscores the notion that societal factors could impede optimal infant feeding practices, even when mothers are well educated or have stable financial resources.

On another hand, some global and regional literature shows that higher maternal education is often associated with better breastfeeding and complementary feeding practices [45–47]. Similarly, Khalaf, Mathew [21] demonstrated that social development and generational changes in Oman have transformed breastfeeding practices, often benefiting women from higher socioeconomic backgrounds. However, these associations mainly focus on breastfeeding practices, which have been extensively studied, rather than complementary infant feeding practices. It is worth noting that the link between breastfeeding and higher maternal education does not necessarily hold when examining complementary feeding practices. Therefore, more research should explore the factors associated with specific complementary infant feeding practices.

This study revealed that adverse birth-related circumstances, such as cesarean delivery, are linked to poorer infant feeding practices. It is widely recognized that vaginal deliveries are associated with healthier infant feeding practices, including early breastfeeding [48, 49]. Reports from the United Nations International Children’s Emergency Fund indicate that vaginal deliveries double the rates of early breastfeeding initiation compared to cesarean sections. Therefore, the circumstances of cesarean deliveries reflect important risks that necessitate special attention and support for infant feeding counseling and follow-up. In fact, these circumstances are often associated with longer hospital stays, which makes them a target for increasing the chances of receiving counseling on infant feeding.

Taken together, these findings reinforce the critical role of healthcare systems in ensuring that mothers receive structured, culturally sensitive counseling to support optimal infant feeding [8, 50]. This study suggests that Oman’s counseling coverage accounts for 43.2%, highlighting the need for increased access to infant feeding counseling. Mobile applications are a valuable resource that enhance maternal knowledge about infant feeding, particularly among younger and busy mothers [51].

### Strengths and limitations

This research benefits from a large, multi-governorate health center–based sample, which enhances the applicability of its results throughout Oman. Additionally, it is one of the few studies in the MENA region that quantitatively investigates the pattern and factors influencing infant feeding practices and beliefs. However, some limitations should be noted. The cross-sectional nature of the study limits the ability to establish causal relationships. The convenience sampling approach and exclusion of two governorates may limit national generalizability. Moreover, since the data is self-reported, there may be a risk of social desirability bias. In addition, recall bias is possible because mothers of children aged one to 3 years were asked to report feeding practices during the first year of life. Finally, the IFB model explained a small portion of the outcome variance, suggesting that additional associating factors warrant investigation.

### Implications

The findings of this study have several implications for practice and future research in Oman and similar settings. Health systems need to prioritize equitable access to comprehensive counseling services, not merely breastfeeding. Incorporating nutrition counseling into routine antenatal and postnatal care, may serve as an effective strategy. Additionally, infant feeding campaigns should adopt culturally sensitive approaches that acknowledge local traditions while correcting misconceptions. Collaboration with community leaders, who often influence infant feeding decisions, may enhance the effectiveness of these initiatives.

Future research in Oman and comparable settings should employ longitudinal designs to explore the causal pathways linking maternal education, income, and counseling with infant feeding beliefs and practices. It is also important to investigate the role of fathers and extended family members, who often influence feeding decisions but remain understudied in this context.

### Conclusions

This study highlights critical determinants to healthy infant feeding among mothers in Oman. Mothers who received infant feeding counseling exhibited higher adherence to the recommendations. Conversely, mothers with high educational attainment or family income demonstrated lower adherence to healthy infant feeding practices. These findings highlight the need for further exploration of mediating sociocultural influences on infant feeding among educated mothers, such as workplace infant-feeding policies and parental leave. Clinically, healthcare providers should enhance access to tailored, structured infant feeding counseling, emphasizing its critical role in promoting optimal feeding practices.
