# Supplementary material for: Maternal determinants of healthy infant feeding practices in Oman: an analytical epidemiology study
Source: Int J Public Health. 2026 Oct 29;71:1609924. doi: 10.3389/ijph.2026.1609924 (PMC13757968; doi:10.3389/ijph.2026.1609924)
Supplement: Supplementary file 1 [file Table1.docx]

| **Supplementary 1. Sample Size Distribution Across Different Governorates** | | | | |
| --- | --- | --- | --- | --- |
| **Governorate** | **0-4 years** | **0.5-1%** | **n sample** | **% sample** |
| 1. North Al Batinah | 82,620 | 413- 826 | 464 | 0.6 |
| 2. Muscat | 70,182 | 351- 702 | 265 | 0.4 |
| 3. Al-Dakhiliyah | 55,815 | 279- 558 | 624 | 1.1 |
| 4. South Al Batinah | 53,737 | 269- 537 | 628 | 1.2 |
| 5. South Al Sharqiyah | 34,797 | 174- 348 | 329 | 0.9 |
| 6. Dhofar | 31,007 | 155- 310 | 97 | 0.3 |
| 7. North Al Sharqiyah | 29,563 | 148- 296 | 371 | 1.3 |
| 8. Al-Dhahirah | 23,616 | 118- 236 | 291 | 1.2 |
| 9. Al-Buraimi | 9,860 | 49- 99 | 150 | 1.5 |
| **Total** | 399,353 | 1956- 3912 | 3219 | 0.8 |
