# Supplementary material for: Maternal determinants of healthy infant feeding practices in Oman: an analytical epidemiology study
Source: Int J Public Health. 2026 Oct 29;71:1609924. doi: 10.3389/ijph.2026.1609924 (PMC13757968; doi:10.3389/ijph.2026.1609924)
Supplement: Supplementary file 2 [file Table2.docx]

| **Supplementary 2. Detailed Description of IFP.** | | |
| --- | --- | --- |
|  | **n** | **%** |
| Q1. Exclusive breastfeeding in the first six months. |  |  |
| Never | 195 | 6.4 |
| Rarely | 337 | 11.1 |
| Sometimes | 513 | 16.9 |
| Mostly | 905 | 29.8 |
| Always | 1086 | 35.8 |
| Q2. Breastfeeding in the first year. |  |  |
| No | 353 | 11.6 |
| Yes | 2683 | 88.4 |
| Q3. Infant formula after six months. |  |  |
| Less than 6 months | 1376 | 45.3 |
| 6-11 months | 743 | 24.5 |
| 12 or more months | 214 | 7.0 |
| Not yet | 703 | 23.2 |
| Q4. Solid foods after six months. |  |  |
| Less than 4 months | 78 | 2.6 |
| 4-5 months | 823 | 27.1 |
| 6 or more months | 2090 | 68.8 |
| Not yet | 45 | 1.5 |
| Q5. Meat, chicken, and fish after six months. |  |  |
| Less than 4 months | 24 | .8 |
| 4-5 months | 213 | 7.0 |
| 6 or more months | 2595 | 85.5 |
| Not yet | 204 | 6.7 |
| Q6. 100% fresh, unsweetened juice after first year. |  |  |
| Less than 6 months | 326 | 10.7 |
| 6-11 months | 1633 | 53.8 |
| 12 or more months | 756 | 24.9 |
| Not yet | 321 | 10.6 |
| Q7. Yogurt after six months. |  |  |
| Less than 6 months | 594 | 19.6 |
| 6-11 months | 1902 | 62.6 |
| 12 or more months | 427 | 14.1 |
| Not yet | 113 | 3.7 |
| Q8. Egg after six months. |  |  |
| Less than 6 months | 174 | 5.7 |
| 6-11 months | 1587 | 52.3 |
| 12 or more months | 843 | 27.8 |
| Not yet | 432 | 14.2 |
| Q9. Fresh cow’s milk after first year. |  |  |
| Less than 6 months | 77 | 2.5 |
| 6-11 months | 633 | 20.8 |
| 12 or more months | 1491 | 49.1 |
| Not yet | 835 | 27.5 |
| Q10. Honey after first year. |  |  |
| Less than 6 months | 190 | 6.3 |
| 6-11 months | 536 | 17.7 |
| 12 or more months | 1394 | 45.9 |
| Not yet | 916 | 30.2 |
